# Chemical and Biochemical Characterization of Essential Oils and Their Corresponding Hydrolats from Six Species of the *Lamiaceae* Family

**DOI:** 10.3390/plants10112489

**Published:** 2021-11-17

**Authors:** Cristina Laura Popa, Andreea Lupitu, Maria Daniela Mot, Lucian Copolovici, Cristian Moisa, Dana Maria Copolovici

**Affiliations:** 1 Biomedical Sciences Doctoral School, University of Oradea, 1 University St., 410087 Oradea, Romania; cristina8.popa@gmail.com (C.L.P.); mt_dana@yahoo.com (M.D.M.); 2 Faculty of Food Engineering, Tourism and Environmental Protection, Institute for Research, Development and Innovation in Technical and Natural Sciences, Aurel Vlaicu University, Elena Dragoi St. No. 2, 310330 Arad, Romania; pag.andreea@yahoo.com (A.L.); lucian.copolovici@uav.ro (L.C.); dana.copolovici@uav.ro (D.M.C.)

**Keywords:** antioxidant activity, bioactive compounds, by-product valorization, essential oils, GC-MS, hydrolats

## Abstract

Many plants belonging to the *Lamiaceae* family are rich in essential oils (EOs) which are intensively used for aromatherapy, food and beverage flavoring, alternative medicine, cosmetics, and perfumery. Aerial parts of *Thymus vulgaris* L., *Thymus pannonicus* All., *Lavandula angustifolia* L., *Lavandula x intermedia*, *Origanum vulgare* L., and *Origanum vulgare* var. *aureum* L. were subjected to hydrodistillation, and both resulting fractions were analyzed. The purpose of this study was to determine the chemical composition, antioxidant activity, and total phenolic content of six essential oils and their corresponding hydrolats (HDs) through GC-MS and spectrophotometric analyses. Overall, 161 compounds were identified, some found exclusively in essential oils and others in hydrolats, making them individual products with specific end purposes. The total phenolic content was the highest for the *Thymus vulgaris* L. EOs (3022 ± mg GAE L^−1^), because of its high phenolic oxygenated monoterpenes content (thymol and carvacrol) and the smallest for the *Lavandula angustifolia* L. EOs (258.31 ± 44.29 mg GAE L^−1^), while hydrolats varied from 183.85 ± 0.22 mg GAE L^−1^ for *Thymus vulgaris* L. HD and 7.73 mg GAE L^−1^ for *Thymus pannonicus* All. HD. Significant antioxidant effects determined through DPPH^•^ and ABTS^•+^ assays were also observed in samples with higher hydrophilic compounds. The highest antioxidant activity was determined for *Thymus vulgaris* L. EO and its corresponding HD. Although EOs are the principal traded economic product, HDs represent a valuable by-product that could still present intense antiseptic activities, similar to their corresponding EOs (thyme and oregano), or have multiple aromatherapy, cosmetics, and household applications (lavender and lavandin).

## 1. Introduction

Medicinal and aromatic plants have always served as sources of compounds with bioactive properties for ritual, food flavoring, medicinal, cosmetic, and hygienic purposes [1]. These compounds are secondary metabolites which serve many functions in plants, e.g., from signaling to defense molecules, improving the plant’s chances of survival when faced with unfavorable environmental conditions [2]. Essential oils are mostly composed of highly complex, volatile organic compounds which are insoluble in water, mainly composed of monoterpenes and sesquiterpenes [3], representing one of the four main biological classes of natural compounds alongside polyphenols, alkaloids, and glycosides [1,4]. Although essential oils (EOs) have been used for centuries, some cultures still use them for their therapeutic potential as antiseptics, antioxidants and antivirals [5,6,7,8], for ecological agriculture, e.g., as pesticides, and as repellents for insects and mites [9]. One by-product of essential oils is hydrolats (HDs), which consist primarily of water containing less than 1% hydrophilic bioactive substances [3].

Many *Lamiaceae* family plants have a high EO content [10], primarily in their leaves and flowers, but also in some fruits and seeds [11]. The total economic value of the essential oil industry worldwide in 2007 was around 2.00 billion USD [12,13,14], and is expected to reach 27 billion USD by 2022 [15], from which lavender essential oil represents approximately 45 million USD [16].

*Thymus vulgaris* L. is an aromatic perennial subshrub, native to the southern regions of Europe [6,17], rich in terpenoids (thymol, carvacrol, linalool, geraniol, *p*-cymene, 𝛾-terpinene, limonene, *β*-caryophyllene), phenolic compounds (quinic acid, rosmarinic acid, caffeic acid, *p*-coumaric acid, syringic acid) and flavonoids (apigenin, luteolin, cirsimaritin) [17]. Thyme bioactive compounds have many pharmacological effects: antioxidant [18,19], antimicrobial, antifungal, anxiolytic, anticancer, antiviral, anti-inflammatory, neuroprotective, lipolytic, cardio- and hepato-protective [19,20,21,22].

*Thymus pannonicus* All. is a perennial creeping subshrub in Central and Eastern Europe (also known as Hungarian or Eurasian thyme) that prefers open fields, prairies and rocky areas terrains. Infusions rich in penolic compounds present antimicrobial and antioxidant properties [23], being intensively used in oral hygiene products like mouth washes and gargles for cold, and cough relief [24]. Depending on its chemotype, the EO has high amounts of *β*-citral, geranial, thymol, or carvacrol, alongside *p*-cymene, 𝛾-terpinene, linalool [23,24]. 

*Lavandula angustifolia* L. is indigenous to the Mediterranean region [25,26], and nowadays has worldwide distribution and represents an important commercial essential oil crop [26,27]. The main characteristic of lavender EO is its higher content in linalool/linalyl acetate and low camphor content [1,12,27,28,29]. It is usually used in perfumery and cosmetic products, while lavandin EO with a higher camphor percentage is used in household cleaning products [30]. Lavender EO yield is around 3% [31]; it has many applications as complementary and alternative medicine (CAM) [32,33,34], with numerous applications in mental health settings as an antidepressant, sedative, anxiolytic, and neuroprotective [32], as well as showing antiseptic, antihypertensive, antispasmodic, analgesic and anti-inflammatory properties [27,33,35]. 

*Lavandula x intermedia* (lavandin) is a natural hybrid with a high EO yield, resulting from interbreeding *L. angustifolia x L. latifolia*. It is a relatively tall evergreen shrub with an average height ranging from 60 to 150 cm, with greyish leaves and long fragrant violet inflorescences that bloom by the end of June to July [1,36,37]. The main EO components are 1.8-cineole, linalool, camphor, isoborneol alongside linalyl acetate and lavandulyl acetate [29,30]. Having a slightly different odor, lavandin EO is rarely used in perfumery and pharmaceuticals, but is widely used in household hygiene products (detergents and other cleaning products, insecticides) [12]. 

*Origanum vulgare* L. is native to Southern Europe in the Mediterranean region. It is mainly found as a perennial shrub with an essential oil content of around 2% *v*/*w*. The EO is mainly composed of monoterpenoids, giving it its specific, pungent smell (𝛾-terpinene, *p*-cymene, thymol, and carvacrol). Its EO presents intense pharmacological activities (antibacterial, antifungal, anticancer, anti-inflammatory, antioxidant) [8,11,38,39,40] and has a high bicyclic monoterpenoid content (*⍺*-thujene, sabinene, germacrene D) [41,42]. 

*Origanum vulgare* var. *aureum* L., commonly known as golden oregano, is a tall wood perennial plant up to 50 cm in height with bright green and golden-yellow leaves. It forms clusters of white flowers which are used for culinary, pharmaceutical, and decorative purposes [43]. Its main EO constituents are linalool, *p*-cymene, *γ*-terpinene, presenting potent antioxidant, antibacterial and antifungal activities [44]. 

Despite the fact that the chemical compositions of EOs have been well studied, few research papers have focused on the aqueous phase, i.e., the by-product resulting from the EO industry known as HDs or floral water [45]. The water-soluble EO components dissolved in the distillation water give the resulting HD its characteristic scent and flavor [36,46]. Most HDs have no further applications because of the low abundance of compounds of interest, and as such, are generally discarded [1,36]. Nonetheless, they are sometimes used in the food industry as flavoring agents (deserts and beverages), and in the cosmetic sector in skincare products [47]. 

This study had the following objectives: (i) to broaden the limited existing data regarding the chemical compositions of several EOs and their corresponding HDs using GC-MS analysis; and (ii) to perform biochemical analysis to determine the antioxidant activity and the total phenolic content of the EOs and their correspondent HDs. 

## 2. Results

### 2.1. Essential Oils and Hydrolats Chemical Composition 

**Figure 1:**
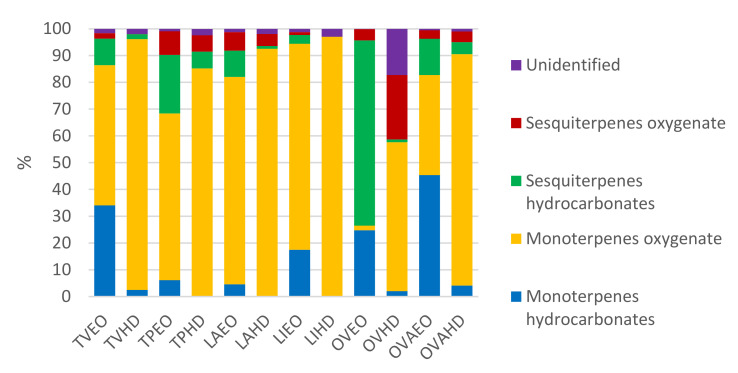
Major compound classes present in all EOs, and HDs determined through GC-MS (EOs are named TVEO, TPEO, LAEO, LIEO, OVEO, OVAEO from *Thymus vulgaris* L., *Thymus pannonicus* All., *Lavandula angustifolia* L., *Lavandula x intermedia* L., *Origanum vulgare* L., *Origanum vulgare* var. *aureum* L., respectively; and HDs are named TVHD, TPHD, LAHD, LIHD, OVHD, OVAHD from *Thymus vulgaris* L., *Thymus pannonicus* All., *Lavandula angustifolia* L., *Lavandula x intermedia* L., *Origanum vulgare* L., *Origanum vulgare* var. *aureum* L., respectively).

Table 1 presents the chemical composition of six EOs and their corresponding concentrated HDs resulting from the same distillation process. In total, 161 compounds were identified, of which 54 were specific to the EOs, 66 were commonly found in both EOs and HDs, and 41 were found exclusively in HDs. The major classes of compounds are presented in Figure 1. 

Considering that distillation is still the most used method for obtaining EOs, many HDs are generated in the process as by-products. These HDs have low concentrations of bioactive compounds, i.e., usually under 1000 mg per litter [48,49], while still presenting antioxidant and antimicrobial effects.

**Figure 2:**
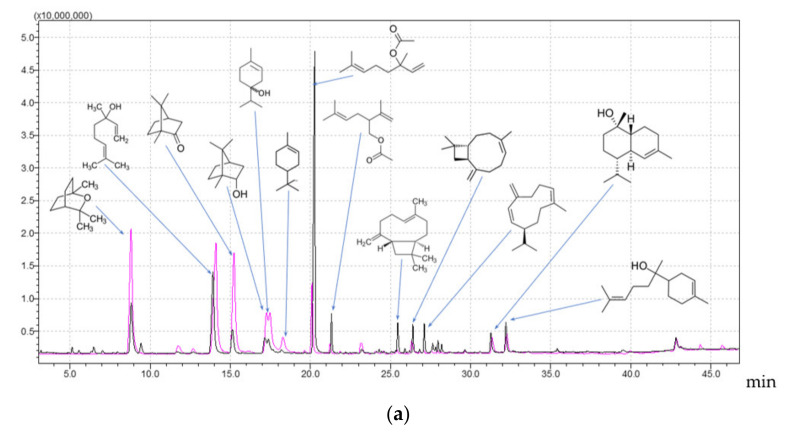

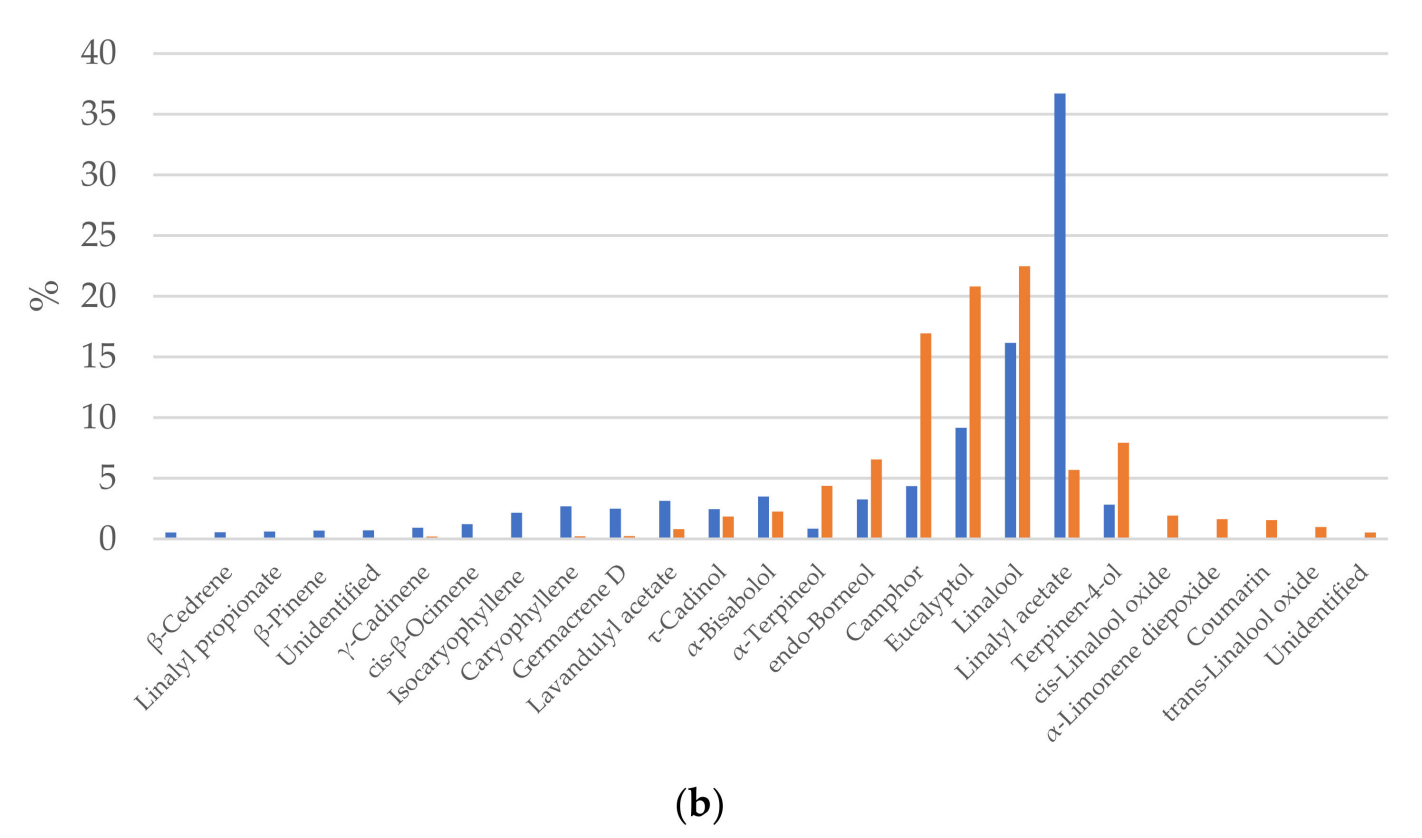
Overlayed chromatograms for LAEO (black) and LAHD (pink) (**a**) and a comparison between the major compounds with a concentration above 0.5%, where LAEO is with blue and LAHD with orange (**b**).

Comparing the chemical profiles of the EOs and HDs resulting from the same distillation batch (Figure 2a) showed that both products had common compounds in different ratios. However, they both contained unique compounds and should be considered independent products. In Figure 2b, for lavender EO, we considered only constituents with concentrations above 0.5%. We compared 20 components, of which 14 were shared with their corresponding HD, and only five compounds with a concentration above 0.5% were exclusively found in the HD.

**Figure 3:**
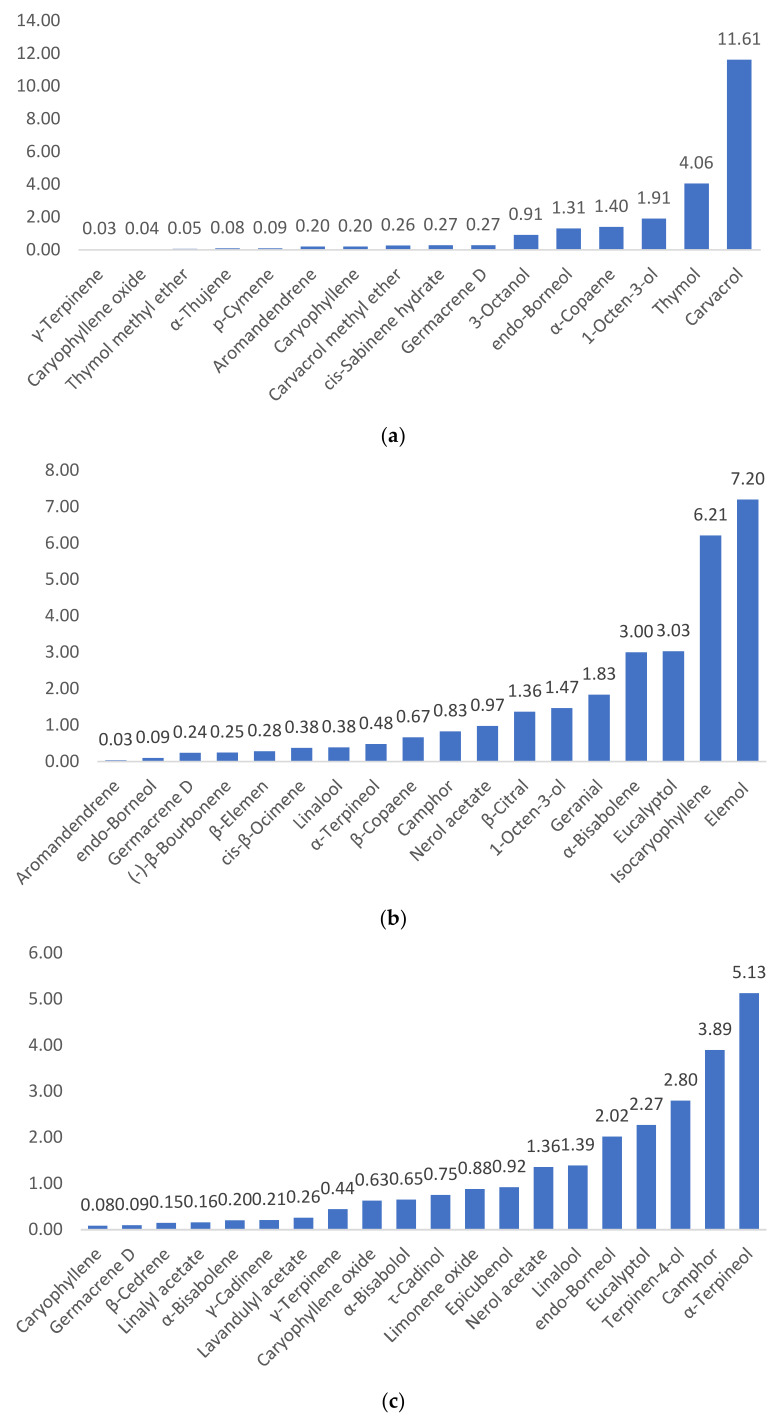

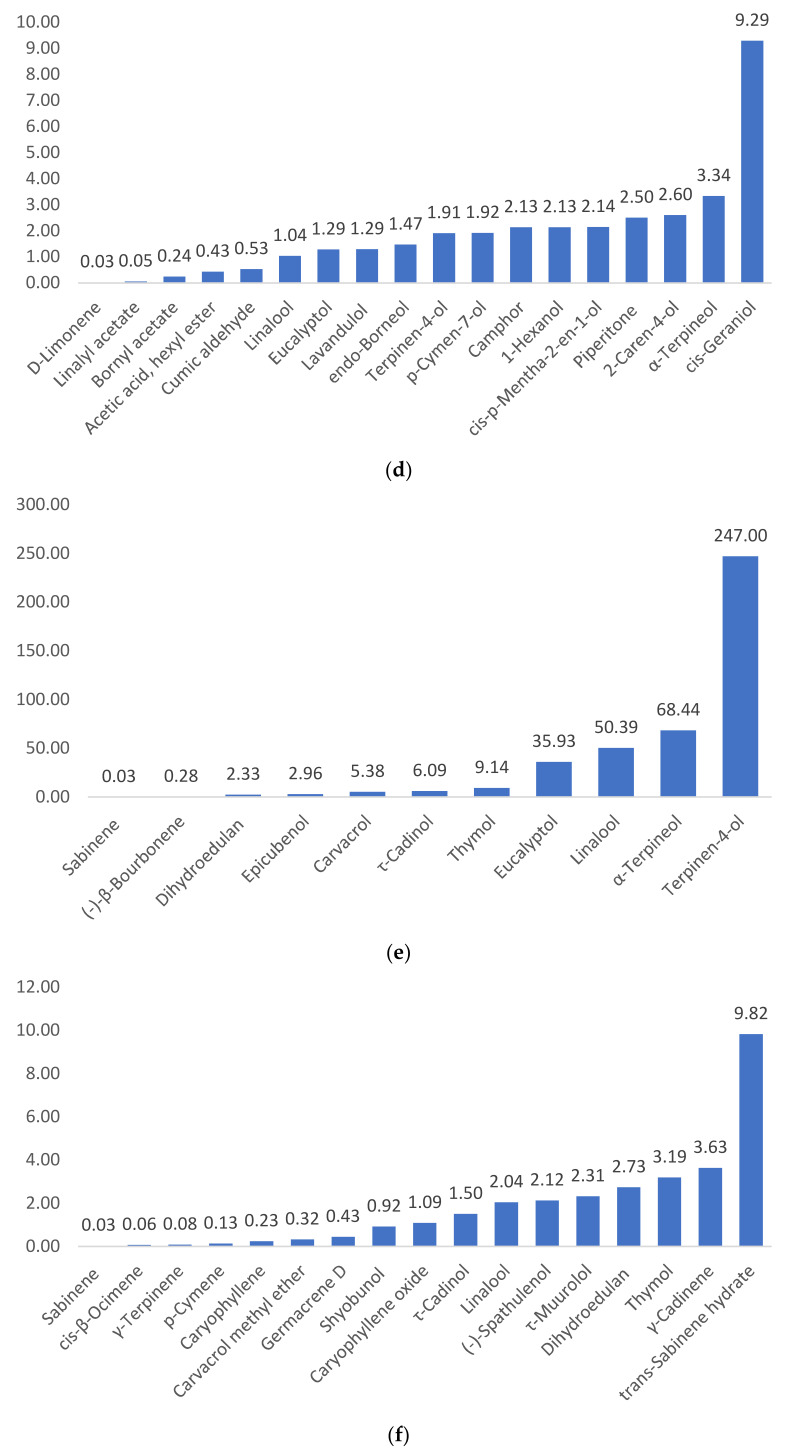
Ratios of compounds in hydrolats to essential oils: (**a**) TVHD/TVEO, (**b**) TPHD/TPEO, (**c**) LAHD/LAEO, (**d**) LIHD/LIEO, (**e**) OVHD/OVEO, (**f**) OVAHD/OVAEO.

To further compare the chemical compositions of the EOs and HDs in the analyzed samples, we determined the ratios of commonly found compounds in both samples. The results are shown in Figure 3.

### 2.2. Biochemical Analysis of the Essential Oils and Hydrolats

#### 2.2.1. Total Phenolic Content

Folin-Ciocâlteu reagent was used to determine the total phenolic content of the EO and HDs samples. For the EO, a 1:10 dilution with methanol was needed, while the HD was used without dilution. The results varied between 3022 mg GAE L^−1^ for TVEO and 7.73 mg GAE L^−1^ for TPHD.

The data are presented in Table 2.

#### 2.2.2. Antioxidant Activity

For all EOs and HDs, the antioxidant activity was evaluated using DPPH^•^ and ABTS^•+^ assays (Table 3). The inhibition results ranged from 4.89% for LIHD to 94.27% for TVEO for DPPH^•^ assay and from 10.11% for TPHD to 98.38% for LAEO for ABTS^•+^ assay.

## 3. Discussions

The plants in this study were chosen based on two criteria: (i) intense usage for their strong antiseptic activities (thyme [20,21,22,23] and oregano [8,11,38,39,40]), and (ii) aromatherapy, cosmetic, and household purposes (lavender [26,27] and lavandin [12]).

In several countries, the production of EOs is one of the most important industries, with a trading market worth billions of USD annually. It has been projected that the EO market will reach 27 billion USD in 2022. The majority of HDs are wasted, and the recovery of this by-product could be economically valued.

The chemical composition of EOs and HDs were determined using the GC-MS technique, which is a gold standard in the field, allowing the determination of all major and minor compounds. The average chemical composition of EOs comprised two major compound classes: hydrocarbonated compounds or terpenes (monoterpenes, sesquiterpenes, diterpenes) and oxygenated compounds or terpenoids (derived from terpenes, alcohols, aldehydes, phenols, esters, ketones, lactones) [50].

Hidrocarbonated terpenes (limonene, *β*-caryophyllene, pinenes) are nonpolar and do not bond with water molecules. Therefore, they are found almost exclusively in EOs and rarely in HDs; their presence in the latter usually indicates poor separation [50].

In contrast, HDs are rich in many oxygenated compounds which are more soluble in water, making thyme, oregano, and lavender HDs rich in biologically active compounds, like terpenes, compared to citrus or coniferous HDs [50].

According to Šilha et al. [3], the abundance of compounds determined in the HDs results from the favorable conditions present during the steam distillation process instead of hydrodistillation. Some compounds can interact with the surrounding boiling water and get transformed into different compounds through oxidation, polymerization, or hydrolyzation. (e.g., *β*-caryophyllene to caryophyllene oxide, limonene to limonene oxide) [51].

According to Garneau et al. [48], *Melissa officinalis* has around 30 compounds found specifically in its EO, 24 in the HD, among which 11 are commonly found in both products.

In the wild thyme samples analyzed in this study, i.e., TVEO and TVHD, 57 and 19 compounds respectively were identified, of which three were exclusively found in the HD. The chemical profiles were represented mainly through oxygenated monoterpenes 52.4% in TVEO and 93.68% in TVHD, represented by thymol methyl ether, carvacrol methyl ether, thymol, carvacrol, followed by hydrocarbonated monoterpenes comprising 34.02% in TVEO and 2.47% in TVHD, represented by *p*-cymene. Other studies have indicated that thymol, *p*-cymene, limonene, and carvacrol are the major terpenes detected [11,52]. Sesquiterpenes were almost exclusively identified in TVEO, with 11.96% instead of 1.91% being determined in TVHD.

Usually, the chemical compositions of HDs are different from those of their corresponding EOs, being enriched in hydrophilic oxygenated terpenes, especially phenols [53].

Because of their phenolic structure, thymol 62.96% and carvacrol 21.48% were present in high quantities in TVHD, as previously reported in other studies [53,54].

Hungarian thyme [23,24,55] revealed a citral chemotype with a similar oxygenated monoterpenes pattern, i.e., 62.26% in TPEO and 85.21%, with *β*-citral, geranial, and thymol being the major compounds. In TPEO, high levels of sesquiterpenes were identified, with germacrene D and nerolidyl acetate being the major compounds. TPHD presented 28 exclusive compounds, mainly oxygenated monoterpenes.

GC-MS analysis revealed 42 and 39 chemical constituents respectively in lavender samples, while identifying 98.75% for LAEO and 98.03% for LAHD of all compounds. Oxygenated monoterpenes represented the major class of compounds in LAEO, accounting for 77.43% (linalyl acetate 36.7%, linalool 16.15%, and eucalyptol 9.16%), followed by sesquiterpenes hydrocarbons 9.84%, oxygenated sesquiterpenes 6.91%, and monoterpenes hydrocarbon 4.57% [56]. As expected, in LAHD, almost 97% of all identified compounds were in the oxygenated form, and seven were not detected in the essential oil, but were found only in the HD. Oxygenated monoterpenes accounted for 92.51% (linalool 22.47%, eucalyptol 20.8%, camphor 16.94%, terpinen-4-ol 7.92%, *α*-terpineol 4.36%), while oxygenated sesquiterpenes represented 4.46%. As previously reported, the presence of eucalyptol and camphor indicates contamination with lavandin [29,30]. Sesquiterpene hydrocarbons accounted for only 1.02%, and their influence within the biochemical analysis was negligible.

Even if lavandin EO is considered to be of lower quality than lavender EO, it has gained popularity due to increasing demand and its higher EO production yield [10]. Therefore, for lavandin, 56 and 36 compounds were identified, respectively, accounting for 98.75% in LIEO and 97.09% in LIHD. The same pattern was observed for LIEO as for LAEO, i.e., 76.93% of the identified compounds were oxygenated monoterpenes (linalool 28.77%, eucalyptol 18.97%, endo-borneol 9.3%, terpinen-4-ol 5.48%, camphor 4.32%, and only a small amount of linalyl acetate 3.73%). For LIHD, essential oil separation was optimally performed, and 16 exclusive compounds were identified. Oxygenated monoterpenes accounted for 96.86% of all the identified compounds (linalool 29.83%, eucalyptol 24.38%, endo-borneol 13.65%, terpinen-4-ol 10.45%, camphor 9.21%).

In oregano samples, a different pattern emerged [57]. In OVEO the major class of compounds was represented by hydrocarbonated sesquiterpenes and monoterpenes accounting for 69.2% and 24.73% respectively, with high quantities of germacrene-D 22.63%, sabinene 16.45%, *α*-farnesene 15.64%, isocaryophillene 13.41%, bicyclogermacrene 5.18%, and *trans*-*β*-ocimene 4.96%. Oxygenated monoterpenes 1.75% and sesquiterpenes 4.21% were represented by germacren-D-4-ol 2.97% as major compounds.

In OVHD, 21 compounds were specific to the HD. Oxygenated monoterpenes and sesquiterpenes were the major compounds classes, accounting for 55.63% and 24.03%, with high levels of 1-octen-3-ol 13.31%, caryophyllene oxide 12.44%, linalool 11.59%, *α*-terpineol 6.16%, (-)-spathulenol 5.64%, eucalyptol 5.39%, and terpinen-4-ol 4.94%. Hydrocarbonated and oxygenated monoterpenes and sesquiterpenes were detected under 4%. OVHD had the highest quantity of unidentified compounds 17.29%.

In golden oregano samples, hydrocarbonated and oxygenated monoterpenes represented the major compound groups accounting for 82.73% in OVAEO and 90.51% in OVAHD. In accordance with previous studies [44], in OVAEO the major compound was linalool 26.54% followed by *p*-cymene 20.81%, *γ*-terpinene 13.73% and thymol 6.88%. In OVAHD, eight compounds were specific, while the major compounds were linalool 54.09%, and thymol 21.96%.

The ratio of hydrolats to essential oils has been calculated for the major common compounds that occur in both sample types (Figure 3). These results confirmed, once again, the presence of oxidized compounds as major compounds in the corresponding HDs. For example, the highest ratio values for one of the compounds found in HDs compared with EOs is carvacrol 11.61 for TVHD/TVEO; elemol 7.20 for TPHD/TPEO; α-terpineol 5.13 for LAHD/LAEO; cis-geraniol 9.29 for LIHD/LIEO; terpinen-4-ol 247 for OVHD/OVEO; trans-sabinene hydrate (4-thujanol) 9.82 for OVAHD/OVAEO.

Overall, a similar chemical pattern was observed for all EOs and their corresponding HDs, polarity influencing the distribution of chemical compounds. The absence of oxygen in the nonpolar molecules like hydrocarbonated monoterpenes and sesquiterpenes (*p*-cymene, *γ*-terpinene, germacrene D, aromandendrene), make these compounds specific to the EO, and their presence in the HDs could indicate a poorly performed separation.

Instead, slightly more water-soluble compounds, like oxygenated monoterpenes and sesquiterpenes (alcohols: linalool, terpinen-4-ol, eucalyptol, geraniol; ketones: camphor, piperitone, D-carvone; aldehydes: *β*-citral, geranial; phenols: thymol, carvacrol) are usually present in higher quantities, and the higher the polarity, the higher the proportion of dissolved compounds in HDs. Therefore, a difference between the EO and its corresponding HD is visible, and both products need to be considered independent of each other to recommend them for different purposes.

The total phenolic content among all six EOs samples varied as follows: TVEO > OVAEO > TPEO > OVEO > LIEO > LAEO and for their six corresponding HDs TVHD > OVAHD > LIHD > LAHD > OVHD > TPHD. As previously reported by other studies [52,56], thyme plants have high amounts of thymol and carvacrol, both of which are phenolic oxygenated monoterpenes, leading to a high total phenolic content. The lowest phenolic equivalent for EOs was observed in LAEO with 258.31 mg GAE L^−1^, while the highest was recorded for TVEO with 3022.36 mg GAE L^−1^.

Among the HDs, TVHD has the highest phenolic content, i.e., 183.85 mg GAE L^−1^ in accordance with its chemical composition, thymol, and carvacrol, accounting for over 80% of its constituents. In comparison, TPHD and OVHD presented the lowest phenolic content, i.e., 7.7 mg GAE L^−1^ following their low oxygenated terpenes content.

The antioxidant activity of the EOs and HD samples was evaluated using DPPH^•^ and ABTS^•+^ assays. The antioxidant activity for all six EOs and their corresponding HDs was influenced mainly by the presence of *p*-cymene, *γ*-terpinene, eucalyptol, linalool, thymol, carvacrol, and the synergistic role that the combination of one or more compounds played was reported previously by other studies [58].

The antioxidant activity determined through the DPPH^•^ assay showed that TVEO and OVEO had a higher inhibition, i.e., 94.27% and 89.11% respectively, compared to the rest of the EOs taken into this study: TVEO > OVEO > OVAEO > TPEO > LIEO > LAEO. In the case of the HDs, only TVHD had a relatively high DPPH^•^ inhibition (16.97%) compared to the other samples (over 5%): TVHD > OVAHD > TPHD > LAHD > OVHD > LIHD.

Through the ABTS^•+^ assay, almost all EOs samples presented a high inhibition, with an average of 87%: LAEO > OVAEO > TVEO > LIEO > TPEO > OVEO. For HDs, only TVHD and OVAHD presented values over 70%, the others averaged around 11%, and the results were calculated as follows: TVHD > OVAHD > TPHD > LAHD > OVHD > LIHD.

As presented by other studies [59,60,61], the more hydrophilic compounds present in the samples (EOs and HDs) were better reproduced by the ABTS^•+^ assay than the DPPH^•^ assay, which is more sensitive for samples containing phenolic compounds and derivates. The data suggested that using the ABTS^•+^ assay, we obtained higher values of the antioxidant activity of EOs and HDs compared to DPPH^•^ assay. The different values of the antioxidant activity measured for the same sample (EO or HD) could be explained by the different mechanisms involved in the reactions of radical antioxidant and sample compounds [62,63].

## 4. Materials and Methods

### 4.1. Plant Material

Fresh herbs of *Thymus vulgaris* L., *Thymus pannonicus* All., *Lavandula angustifolia* L., *Lavandula x intermedia* L., *Origanum vulgare* L., and *Origanum vulgare* var. *aureum* L. were obtained from a local producer in Arad County, Romania. Voucher specimens from these plants are deposited at “Aurel Valicu” University of Arad, Romania. All plant materials were air-dried and stored in paper bags before distillation.

#### 4.1.1. Essential Oil and Hydrolat Extraction

Dried aerial parts were submitted to steam distillation using small-scale copper distillation equipment. The resulting EOs and HDs were separated using a separation funnel and stored at +4 °C until further usage.

#### 4.1.2. Hydrolat Liquid-Liquid Extraction (LLE)

The dispersed and dissolved compounds in the HD samples were separated by LLE using a modified method, as described by Paolini et al. [47]. Briefly, 1 mL hexane and 25 mL HD were mixed and sonicated at room temperature, at 35 kHz, for 1 h at 100% power, using the Elmasonic TI-H5 (Elma, Schimdbauer GmbH, Singen, Germany). Subsequently, the hexane-hydrolat mixture was centrifuged for 5 min at 7000 rpm using a Hettich ultracentrifuge (Rotina 380 R, Hettich GmbH, Tuttlingen, Germany), and the organic layer was filtered through a 0.24 µm PTFE syringe filter before GC-MS analysis. This process was performed three times, and the resulting organic extracts were combined.

#### 4.1.3. Annotations

For all the EOs and HDs, the annotations used are presented in Table 4.

### 4.2. Chemical Composition of EOs and HDs Determined by GC-MS

EOs and HDs were analyzed by using a gas chromatograph (GC, Shimadzu 2010, Kyoto, Japan) coupled with a mass spectrometer (MS, TQ 8040, Shimadzu, Kyoto, Japan) using a method described earlier in Moisa et al. [7]. Briefly, the EOs and HDs constituents were determined by a gas chromatograph (Shimadzu2010, Kyoto, Japan) coupled with a triple quadrupole mass spectrometer (TQ 8040, Shimadzu, Kyoto, Japan), and an optima 1MS  +  WAX column (30 m × 0.25 mm i.d., 0.25 µm film thickness, Macherey–Nagel, Duren, Germany). The carrier gas used was He, with a 1 mL min^−1^ flow. The oven temperature was initially 70 °C that was held for 11 min, and raised to 190 °C at a rate of 5 °C min^−1^ and then to 240 °C at a rate of 20 °C min^−1^ where it was kept for 5 min. Injector and MS source temperatures were set to 250 °C and 200 °C, respectively. The injection volume was 1 µL, with a split ratio of 10:1. Before the injection, EOs samples were diluted (1:25, v:v), and the HDs samples were injected as obtained. All the samples were were filtered using 0.45 µm PTFE membrane. All chemical constituents were identified using spectra libraries NIST 14 and Wiley 09 [6,7], compared with some commercial standards (α-pinene; sabinene; β-pinene; β-myrcene; α-phellandrene; 3-carene; D-limonene; cis-β-ocimene; trans-β-ocimene; carvacrol; caryophyllene), and by comparing their retention indices (abbreviated RI), determined relative to the time of retention values of n-alkanes (C10–C35), on capillary columns with those found in the literature [64].

### 4.3. Total Phenolic Content

All samples were analyzed spectrophotometrically for total phenolic content using Folin-Ciocâlteu reagent (FCR) [65], and the results were calculated and expressed as GAE/L (mg gallic acid equivalents). The HDs were analyzed without dilution, adding to 1 mL of sample, 0.5 mL FCR, 2 mL Na_2_CO_3_ 20%, and 5 mL distilled water. The reaction time was 1.5 h in the dark at room temperature. Afterward, the absorbance was recorded at 765 nm against a blank prepared in the same conditions, using a UV-Vis spectrophotometer (Specord 200, Analytik Jena AG, Jena, Germany) [65].

### 4.4. Antioxidant Activity

#### 4.4.1. DPPH^•^ Method

The radical scavenging activity of the EOs and HDs was analyzed using 1,1-diphenyl-2-picrylhidrazyl radical (DPPH^•^) and measured spectrophotometrically at 517 nm after 1 h reaction time in the dark, as described by Moisa et al. [65]. The inhibition was calculated with the following equation:

*Inhibition (%I)* = [(*A*_𝑐𝑜𝑛𝑡𝑟𝑜𝑙_ − 𝐴_𝑠𝑎𝑚𝑝𝑙𝑒_)/𝐴_𝑐𝑜𝑛𝑡𝑟𝑜𝑙_] × 100

where *A_control_* is the absorbance of the DPPH^•^ solution, and *A_sample_* is the absorbance recorded for the mixture of extract and DPPH^•^ solution.

#### 4.4.2. ABTS^•+^ Method

The scavenging activity of the EOs and HDs was analyzed using the 2,2′-azino-bis(3-ethylbenzothiazoline-6-sulphonic acid (ABTS^•+^ radical), following an earlier described method [66]. Briefly, 1 mL ABTS^•+^ was mixed with 0.5 mL sample (EOs, HDs and ultrapure water as control) and measured spectrophotometrically at 734 nm after 10 min reaction in the dark.

### 4.5. Statistical Analysis

All statistical analyses were further conducted with GraphPad Prism version 9.2.0 (San Diego, CA, USA). The resulting data were further analyzed with ANOVA and Tukey’s post hoc test. The significantly different means *p* < 0.05 were marked with different letters.

## 5. Conclusions

In this work, chemical investigations using GC-MS revealed that the same batch of EOs and their corresponding HDs obtained from six plants from *Lamiaceae* family (thyme, lavender, and oregano) had different compositions of bioactive compounds. HDs, as by-products obtained in the distillation of EOs, have a pleasant smell, a natural taste, and, depending on their oxygenated terpenes content, sometimes their smell is superior to that of the corresponding EO.

In our work, DPPH^•^ and ABTS^•+^ assays demonstrated that both EOs and HDs present antioxidant activities, further confirming that HDs could be considered as independent products and with high economic value as flavoring agents in soft drinks and food products, or in aromatherapy, natural cosmetics, or green synthesis applications.

Although EOs are the principal traded economic product, HDs represent a valuable by-product that could present intense antiseptic activities, similar to those of their corresponding EOs (thyme and oregano), or have multiple aromatherapy applications or cosmetic and household uses (lavender and lavandin).

## Figures and Tables

**Table 1:** Chemical composition of the essential oils and hydrolats identified by GC-MS (%).

Nr. Crt.	RI	Compound Name	Class	TV EO	TV HD	TP EO	TP HD	LA EO	LA HD	LI EO	LI HD	OV EO	OV HD	OVA EO	OVA HD
1	930	*α*-Thujene	MH	0.89	0.07	0.04	-	0.53	-	0.26	-	0.07	-	0.75	-
2	939	*α*-Pinene *	MH	0.68	-	0.21	-	-	-	1.31	-	0.17	-	0.31	-
3	954	Camphene	MH	0.69	-	0.23	-	0.22	-	0.73	-	-	-	0.06	-
4	975	Sabinene *	MH	0.13	-	0.28	-	0.21	-	0.66	-	16.45	0.44	1.32	0.04
5	979	*β*-Pinene *	MH	0.31	-	2.19	-	0.69	-	1.48	-	0.65	-	1.31	-
6	990	*β*-Myrcene *	MH	-	-	0.07	-	0.24	-	-	-	-	-	-	-
7	1002	*α*-Phellandrene *	MH	-	-	-	-	-	-	0.13	-	-	-	0.1	-
8	1003	2-Carene	MH	-	-	-	-	0.39	-	-	-	-	-	-	-
9	1011	3-Carene *	MH	-	-	-	-	0.18	-	0.3	-	1.12	-	-	-
10	1012	(+)-4-Carene	MH	0.75	-	-	-	-	-	0.13	-	0.08	-	1.4	-
11	1014	*α*-Terpinene	MH	-	-	0.11	-	-	-	-	-	-	-	0.09	-
12	1020	D-Limonene *	MH	0.33	-	0.21	-	-	-	2.65	0.07	-	-	-	-
13	1021	*o*-Cymene	MH	-	-	-	-	-	-	0.05	-	-	-	-	-
14	1023	*m*-Cymene	MH	0.1	-	-	-	0.08	-	0.36	-	0.07	-	-	-
15	1024	*p*-Cymene	MH	26.4	2.28	0.07	-	-	-	-	0.16	-	1.09	20.81	2.68
16	1037	*cis*-*β*-Ocimene *	MH	-	-	0.16	0.06	1.21	-	4.92	-	0.6	-	2.71	0.17
17	1050	*trans*-*β*-Ocimene *	MH	-	-	2.14	-	0.13	-	1.58	-	4.96	-	2.57	-
18	1059	*γ*-Terpinene	MH	3.6	0.12	0.32	-	0.09	0.04	0.44	-	0.35	-	13.73	1.15
19	1088	*α*- Terpinolen	MH	0.05	-	0.07	-	-	-	0.64	-	0.04	-	-	0.07
20	1322	Linalyl propionate	MH	-	-	-	-	0.6	-	-	-	-	-	-	-
21		Neo-allo-ocimene	MH	-	-	-	-	-	-	0.28	-	0.17	-	0.19	-
22		*ψ*-Limonene	MH	0.09	-	-	-	-	-	1.56	-	-	-	-	-
23		1-Undecene, 4-methyl-	MH	-	-	-	-	-	-	-	-	-	0.48	-	-
24	873	1-Hexanol	MO	-	-	-	-	-	-	0.3	0.64	-	-	-	-
25	878	4-Hexen-1-ol, (Z)-	MO	-	-	-	-	-	-	-	0.08	-	-	-	-
26	963	3-Octanone	MO	2.83	-	-	-	-	-	-	-	-	2.82	-	-
27	977	1-Octen-3-ol	MO	0.87	1.66	0.3	0.44	-	-	-	0.73	-	13.31	-	1.14
28	978	1-Octen-3-one	MO	-	-	-	0.36	-	-	-	-	-	-	-	-
29	980	3-Octanol	MO	0.9	0.82	-	0.15	-	-	-	-	-	2.59	-	-
30	1010	Acetic acid, hexyl ester	MO	-	-	-	-	-	-	0.14	0.06	-	-	-	-
31	1029	Eucalyptol	MO	-	-	0.37	1.12	9.16	20.8	18.97	24.38	0.15	5.39	-	-
32	1070	*cis*-Sabinene hydrate	MO	2.3	0.62	1.58	-	0.45	-	-	-	-	-	-	-
33	1073	*cis*-Linalool oxide	MO	-	-	-	-	-	1.92	-	0.61	-	-	-	2.79
34	1089	*trans*-Linalool oxide	MO	-	-	-	-	-	0.98	-	0.45	-	-	-	2.18
35	1096	Linalool	MO	-	0.65	0.6	0.23	16.15	22.47	28.77	29.83	0.23	11.59	26.54	54.09
36	1099	*trans*-Sabinene hydrate	MO	0.44	-	-	1.03	-	-	0.37	-	-	0.97	0.11	1.08
37	1105	Propanoic acid, hexyl ester	MO	-	-	-	-	-	-	0.06	-	-	-	-	-
38	1130	2-Pinen-7-one	MO	-	-	-	0.42	-	-	-	-	-	-	-	-
39	1135	Limonene oxide	MO	-	-	-	-	0.34	0.3	-	-	-	-	-	-
40	1138	Limonene epoxide	MO	-	-	-	0.1	-	-	-	-	-	-	-	-
41	1142	*cis* verbenol	MO	-	-	0.86	-	-	-	-	-	-	-	-	-
42	1144	*trans*-Sabinol	MO	0.03	-	-	-	-	-	-	0.09	-	-	-	-
43	1145	*trans*-Verbenol	MO	0.05	-	1.19	-	-	-	-	-	-	-	-	-
44	1146	Camphor	MO	0.04	-	0.46	0.38	4.35	16.94	4.32	9.21	-	1.02	-	-
45	1147	Nopinone	MO	-	-	-	-	-	-	-	0.16	-	-	-	-
46	1148	6-Octenal, 7-methyl-3-methylene-	MO	-	-	-	0.54	-	-	-	-	-	-	-	-
47	1153	Camphene hydrate	MO	-	-	-	-	-	-	-	0.05	-	-	-	-
48	1156	Neryl oxide	MO	-	-	-	0.14	-	-	-	-	-	-	-	-
49	1163	Pinocarvone	MO	-	-	-	-	-	-	-	0.03	-	-	-	-
50	1165	Isoneral	MO	-	-	-	0.7	-	-	-	-	-	-	-	-
51	1168	Lavandulol	MO	-	-	-	-	-	-	0.65	0.84	-	-	-	-
52	1169	endo-Borneol	MO	1.85	2.42	1.58	0.15	3.25	6.55	9.3	13.65	-	1.07	-	-
53	1177	Terpinen-4-ol	MO	0.17	-	0.25	-	2.83	7.92	5.48	10.45	0.02	4.94	0.2	-
54	1184	Isogeranial	MO	0.05	-	-	1.33	-	-	-	-	-	0.25	-	0.13
55	1188	*α*-Terpineol	MO	0.1	-	0.21	0.1	0.85	4.36	0.95	3.17	0.09	6.16	0.19	-
56	1190	Methyl salicylate	MO	-	-	-	-	-	-	-	-	-	-	0.13	-
57	1194	Butanoic acid, hexyl ester	MO	-	-	-	-	-	-	0.86	-	-	-	-	-
58	1199	Estragole	MO	-	-	-	0.25	-	-	-	-	-	-	-	-
59	1206	*cis*-Verbenone	MO	-	-	0.16	-	-	-	-	0.05	-	-	-	-
60	1209	*trans*-Piperitol	MO	-	-	-	-	-	-	-	0.05	-	-	-	-
61	1227	Citronellol	MO	-	-	-	0.28	-	-	-	-	-	-	-	-
62	1232	*cis*-Geraniol	MO	-	-	-	2.18	-	-	0.07	0.65	-	-	-	-
63	1235	Thymol methyl ether	MO	10.78	0.56	-	-	-	-	-	-	0.28	-	-	-
64	1237	Butanoic acid, 2-methyl-, hexyl ester	MO	-	-	-	-	-	-	0.39	-	-	-	-	-
65	1239	*β*-Citral	MO	-	-	20.3	27.7	-	-	-	-	-	-	-	-
66	1241	Isobornyl formate	MO	-	-	-	-	0.07	-	-	-	-	-	-	-
67	1244	Carvacrol methyl ether	MO	6.26	1.64	-	-	-	-	-	-	-	-	3.04	0.97
68	1246	D-Carvone	MO	-	-	-	-	-	-	-	0.15	-	0.18	-	-
69	1252	(-)-*cis*-Myrtanol	MO	-	-	-	0.31	-	-	-	-	-	-	-	-
70	1254	Geraniol	MO	-	-	-	2.03	-	-	-	-	-	-	-	-
71	1255	Piperitone	MO	-	-	-	0.12	-	-	0.04	0.1	-	-	-	-
72	1258	Linalyl acetate	MO	-	-	-	0.28	36.7	5.69	3.73	0.19	0.55	-	-	-
73	1264	(-)-*trans*-Myrtanol	MO	-	-	0.17	-	-	-	-	-	-	-	-	-
74	1268	Geranial	MO	-	-	20.66	37.91	-	-	-	-	-	-	-	-
75	1287	Bornyl acetate	MO	-	0.36	0.2	-	-	-	0.29	0.07	-	-	-	-
76	1290	Thymol	MO	15.52	62.96	9.7	-	-	-	-	-	0.21	1.92	6.88	21.96
77	1292	Lavandulyl acetate	MO	-	-	-	1.99	3.14	0.81	0.99	-	-	-	-	-
78	1299	Carvacrol *	MO	1.85	21.48	0.34	-	-	-	-	-	0.08	0.43	-	0.45
79	1333	Hexyl tiglate	MO	-	-	-	-	-	-	0.15	-	-	-	-	-
80	1352	Thymol acetate	MO	0.05	-	-	-	-	-	-	-	-	-	-	-
81	1381	Nerol acetate	MO	-	-	3.33	3.24	0.14	0.19	0.11	-	-	-	-	-
82	1384	Hexanoic acid, hexyl ester	MO	-	-	-	-	-	-	0.19	-	-	-	-	-
83	1435	Coumarin	MO	-	-	-	-	-	1.54	-	0.13	-	-	-	-
84		*α*-Limonene diepoxide	MO	-	-	-	-	-	1.63	-	-	-	-	-	-
85		*cis*-*p*-Mentha-2-en-1-ol	MO	-	-	-	-	-	-	0.07	0.15	-	-	-	-
86		Linalyl formate	MO	-	-	-	-	-	0.09	0.09	-	-	-	-	-
87		*trans*-Pyranoid linalool oxide	MO	-	-	-	-	-	-	-	0.07	-	-	-	-
88		n-Hexyl butanoate	MO	-	-	-	-	-	-	-	0.05	-	-	-	-
89		(-)-*trans*-Isopiperitenol	MO	-	-	-	-	-	-	-	0.04	-	-	-	-
90		2-Caren-4-ol	MO	-	-	-	-	-	-	0.05	0.13	-	-	-	-
91		Cumic aldehyde	MO	-	-	-	-	-	-	0.47	0.25	-	-	-	-
92		*p*-Cymen-7-ol	MO	0.14	-	-	-	-	-	0.12	0.23	-	-	-	-
93		Car-3-en-5-one	MO	-	-	-	0.12	-	0.32	-	0.07	-	-	-	-
94		*p*-Cymen-8-ol	MO	0.08	-	-	-	-	-	-	-	-	-	-	0.9
95		8,9-Dehydrothymol methyl ether	MO	0.17	-	-	-	-	-	-	-	-	-	-	-
96		3-Carene-2,5-dione	MO	7.9	-	-	-	-	-	-	-	-	-	-	-
97		2,4-Di-tert-butylphenol	MO	-	0.51	-	-	-	-	-	-	-	-	-	-
98		1-Nonen-3-ol	MO	-	-	-	0.12	-	-	-	-	-	0.99	-	-
99		Rosefuran	MO	-	-	-	0.05	-	-	-	-	-	-	0.03	-
100		*p*-Menth-1-en-7-al	MO	-	-	-	0.31	-	-	-	-	-	-	-	-
101		*p*-Menth-3-en-9-ol	MO	-	-	-	0.36	-	-	-	-	-	-	-	-
102		*trans*-3(10)-Caren-2-ol	MO	-	-	-	0.29	-	-	-	-	-	-	-	-
103		Hydroxy-*α*-terpenyl acetate	MO	-	-	-	0.12	-	-	-	-	-	-	-	-
104		Isothymol methyl ether	MO	-	-	-	-	-	-	-	-	0.02	-	-	-
105		Dihydroedulan	MO	-	-	-	-	-	-	-	-	0.12	0.28	0.26	0.71
106		*cis-p*-2-Menthen-1-ol	MO	-	-	-	-	-	-	-	-	-	0.45	-	-
107		3-*p*-Menthen-7-al	MO	-	-	-	-	-	-	-	-	-	0.17	-	-
108		2,4-Di-tert-butylphenol	MO	-	-	-	-	-	-	-	-	-	1.1	-	-
109		Limonene diepoxide	MO	0.02	-	-	0.3	-	-	-	-	-	-	-	-
110		*p*-Cymen-8-ol	MO	-	-	-	-	-	-	-	-	-	-	-	-
111	1376	*α*-Copaene	SH	0.05	0.07	-	-	-	-	-	-	0.15	-	0.26	-
112	1378	isoledene	SH	0.14	-	-	-	0.25	-	0.03	-	-	-	0.43	-
113	1389	(-)-*β*-Bourbonene	SH	0.29	-	1.22	0.3	-	-	-	-	1.65	0.47	0.52	-
114	1391	*β*-Elemen	SH	-	-	0.57	0.16	-	-	-	-	0.51	-	0.21	-
115	1402	Sesquithujene	SH	-	-	-	-	-	-	0.07	-	-	-	-	-
116	1405	*β*-Longipinene	SH	0.03	-	-	-	-	-	-	-	-	-	-	-
117	1408	Isocaryophyllene	SH	-	-	0.24	1.49	2.16	-	2.26	-	13.41	-	3.04	-
118	1414	*cis*-*α*-Bergamotene	SH	0.03	-	0.03	-	0.29	-	0.08	-	1	-	0.11	-
119	1419	*β*-Caryophyllene *	SH	3.14	0.64	0.96	-	2.69	0.22	0.46	-	-	0.32	0.98	0.23
120	1422	*β*-Cedrene	SH	0.1	-	-	-	0.55	0.08	0.04	-	-	-	-	-
121	1432	*β*-Copaene	SH	0.15	-	0.12	0.08	-	-	-	-	0.72	-	0.19	-
122	1440	*α*-Guaiene	SH	0.04	-	-	-	-	-	-	-	-	-	0.46	-
123	1441	Aromandendrene	SH	4.4	0.86	1.97	0.06	-	-	-	-	-	-	-	1.12
124	1444	*β*-Farnesene	SH	-	-	0.32	-	-	-	-	-	-	-	-	-
125	1447	Isogermacrene D	SH	-	-	0.42	-	-	-	-	-	-	-	-	-
126	1454	Humulene	SH	0.13	-	-	-	-	-	-	-	3.08	-	0.22	-
127	1456	*trans*-*α*-Bergamotene	SH	-	-	-	-	-	-	-	-	0.19	-	-	-
128	1459	*γ*-Elemene	SH	-	-	-	-	-	-	-	-	0.37	-	-	-
129	1468	*cis*-Muurola-4(15),5-diene	SH	-	-	-	-	-	-	-	-	1.16	-	-	-
130	1479	*γ*-Muurolene	SH	0.16	-	0.31	-	-	-	0.07	-	0.19	-	0.11	-
131	1481	Germacrene D	SH	1.03	0.28	15.34	3.67	2.49	0.23	0.13	-	22.63	-	5.98	2.59
132	1498	Valencene	SH	0.05	-	-	-	-	-	-	-	-	-	0.16	-
133	1502	Bicyclogermacrene	SH	-	-	-	0.21	-	-	-	-	5.18	-	0.56	-
134	1507	*α*-Farnesene	SH	-	-	0.34	-	0.14	-	0.1	-	15.64	-	-	-
135	1509	*α*-Bisabolene	SH	-	-	0.04	0.12	0.35	0.07	-	-	2.22	-	0.13	-
136	1512	*γ*-Cadinene	SH	0.14	-	-	-	0.92	0.19	0.02	-	0.58	-	0.16	0.58
137	1523	*β*-Sesquiphellandrene	SH	-	-	-	0.15	-	-	-	-	-	-	-	-
138	1544	Nerolidol	SH	-	-	-	-	-	0.23	-	-	-	0.25	-	-
139		Cadina-3,5-diene	SH	-	-	-	-	-	-	-	-	0.52	-	-	-
140	1513	(R)-lavandulyl (R)-2-methylbutanoate	SO	-	-	-	-	-	-	0.33	-	-	-	-	-
141	1561	Nerolidol	SO	-	-	-	1.04	-	-	-	-	-	-	-	-
142	1562	Elemol	SO	-	-	0.6	4.32	-	-	-	-	-	-	-	-
143	1580	Germacren D-4-ol	SO	-	-	0.19	-	-	-	-	-	2.97	-	-	-
144	1582	(-)-Spathulenol	SO	0.29	-	-	0.1	-	-	-	-	-	5.64	0.73	1.55
145	1583	Caryophyllene oxide	SO	1.4	0.06	-	-	0.4	0.25	0.1	-	-	12.44	0.67	0.73
146	1615	Humulene oxide	SO	0.03	-	-	-	-	-	-	-	-	-	0.27	-
147	1631	*γ*-Eudesmol	SO	-	-	-	0.19	-	-	-	-	-	-	-	-
148	1632	Epicubenol	SO	-	-	-	-	0.12	0.11	-	-	0.23	0.68	-	-
149	1640	*τ*-Cadinol	SO	-	-	-	-	2.45	1.84	-	-	0.47	2.86	0.26	0.39
150	1641	Alloaromadendrene oxide-(1)	SO	-	-	-	-	-	-	-	-	-	0.4	0.1	-
151	1642	*τ*-Muurolol	SO	0.06	-	-	-	-	-	-	-	0.21	-	0.16	0.37
152	1644	Aromadendrene oxide-(2)	SO	0.06	-	-	-	-	-	-	-	-	0.4	-	-
153	1648	*α*-Muurolol	SO	-	-	-	-	-	-	-	-	-	1.19	-	-
154	1654	*α*-Eudesmol	SO	-	-	-	0.4	-	-	-	-	-	-	-	-
155	1682	Nerolidyl acetate	SO	-	-	8.03	-	-	-	-	-	-	-	-	-
156	1687	*α*-Bisabolol	SO	-	-	-	-	3.48	2.26	0.65	-	0.33	-	-	-
157	1692	Shyobunol	SO	-	-	-	-	0.46	-	-	-	-	-	0.99	0.91
158	1696	Farnesol	SO	-	-	-	0.08	-	-	-	-	-	-	-	-
159	1762	*cis*-Lanceol	SO	0.17	-	-	-	-	-	-	-	-	-	-	-
160	1765	15-Hydroxy-*α*-muurolene	SO	0.07	-	-	-	-	-	-	-	-	-	-	-
161		Aromadendrane-4,10-diol	SO	-	-	-	-	-	-	-	-	-	0.42	-	-
Total unidentified (%)				1.57	1.94	0.87	2.34	1.25	1.97	1.25	2.96	0.07	17.29	0.57	1.02
Total identified (%)				98.43	98.06	99.13	99.68	98.75	98.03	98.75	97.09	99.93	83.21	99.49	98.98
**Total of Major Compounds (%)**															
Monoterpene hydrocarbonates (MH)				34.02	2.47	6.1	0.06	4.57	0.04	17.48	0.23	24.73	2.01	45.35	4.11
Monoterpenes oxygenate (MO)				52.4	93.68	62.26	85.15	77.43	92.51	76.93	96.81	1.75	55.63	37.38	86.4
Total Monoterpene				86.42	96.15	68.36	85.21	82	92.55	94.41	97.03	26.48	57.64	82.73	90.51
Sesquiterpene hydrocarbonates (SH)				9.88	1.85	21.88	6.24	9.84	1.02	3.26	-	69.2	1.04	13.52	4.52
Sesquiterpene oxygenate (SO)				2.08	0.06	8.82	6.13	6.91	4.46	1.08	-	4.21	24.03	3.18	3.95
Total Sesquiterpene				11.96	1.91	30.7	12.37	16.75	5.48	4.34	-	73.41	25.07	16.70	8.47
Others				1.62	1.94	0.94	2.34	1.25	1.97	1.25	2.96	0.11	17.29	0.57	1.02
Total				100	100	100	100	100	100	100	100	100	100	100	100

RI: calculated retention indices relative to n-alkanes (C10–C35); Compounds identified by using comparison with standards were marked with an asterisk (*).

**Table 2:** Total phenolic content (TPC) analyzed for EO and HDs.

Sample Name	TPC (mg GAE L^−1^)		
Essential Oils		Hydrolats	
TVEO	3022.36 ± 44.29 ^a^	TVHD	183.85 ± 0.22 ^a^
TPEO	846.36 ± 3.44 ^b^	TPHD	7.73 ± 0.01 ^b^
LAEO	258.31 ± 0.53 ^c^	LAHD	10.63 ± 0.03 ^c^
LIEO	355.67 ± 0.20 ^c^	LIHD	12.36 ± 0.01 ^d^
OVEO	681.15 ± 0.39 ^d^	OVHD	7.79 ± 0.03 ^b^
OVAEO	2991.46 ± 27.61 ^a^	OVAHD	38.73 ± 0.02 ^e^

The results are presented as mean ± standard deviation. Superscript letters (a–d) denote significant differences between results on the same data line after Tukey’s test for *p* < 0.05. Means with superscripts having the same letter in the column are not significantly different.

**Table 3:** The antioxidant activity for the EO and HDs determined through DPPH^•^ and ABTS^•+^ assays. Results are depicted as the mean of a triplicate experiment (*n* = 3) ± standard deviation.

Sample Name	DPPH^•^ Assay	ABTS^•+^ Assay
	Inhibition (%)	Inhibition (%)
**Essential oils**		
TVEO	94.27 ± 0.01 ^a^	92.68 ± 0.02 ^a^
TPEO	24.09 ± 0.03 ^b^	87.63 ± 0.02 ^b^
LAEO	9.69 ± 0.06 ^c^	98.38 ± 0.03 ^b^
LIEO	9.73 ± 0.03 ^c^	88.50 ± 0.17 ^c^
OVEO	89.11 ± 0.23 ^d^	53.32 ± 0.76 ^d^
OVAEO	58.18 ± 0.07 ^e^	96.47 ± 0.08 ^e^
**Hydrolats**		
TVHD	16.97 ± 0.07 ^a^	98.16 ± 0.13 ^a^
TPHD	5.47 ± 0.03 ^b^	10.11 ± 0.13 ^b^
LAHD	5.28 ± 0.07 ^b^	12.91 ± 0.11 ^c^
LIHD	4.89 ± 0.03 ^c^	12.76 ± 0.04 ^c^
OVHD	4.92 ± 0.08 ^c^	10.56 ± 0.31 ^b^
OVAHD	6.51 ± 0.08 ^d^	71.36 ± 0.09 ^d^

The results are presented as mean ± standard deviation. Superscript letters (a–d) denote significant differences between results on the same data line after Tukey’s test for *p* < 0.05. Means with superscripts having the same letter in the column are not significantly different.

**Table 4:** Abbreviations for the samples obtained in the present research.

Sample Name	Essential Oil	Hydrolat
*Thymus vulgaris* L.	TVEO	TVHD
*Thymus pannonicus* All.	TPEO	TPHD
*Lavandula angustifolia* L.	LAEO	LAHD
*Lavandula x intermedia* L.	LIEO	LIHD
*Origanum vulgare* L.	OVEO	OVHD
*Origanum vulgare* var. *aureum* L.	OVAEO	OVAHD

## Data Availability

Not available.

## References

[1] Garzoli S., Petralito S., Ovidi E., Turchetti G., Laghezza Masci V., Tiezzi A., Trilli J., Cesa S., Casadei M.A., Giacomello P. (2020). *Lavandula* x *intermedia* essential oil and hydrolate: Evaluation of chemical composition and antibacterial activity before and after formulation in nanoemulsion. Ind. Crops Prod..

[2] Bernardini S., Tiezzi A., Laghezza Masci V., Ovidi E. (2018). Natural products for human health: An historical overview of the drug discovery approaches. Nat. Prod. Res..

[3] Šilha D., Švarcová K., Bajer T., Královec K., Tesařová E., Moučková K., Pejchalová M., Bajerová P. (2020). Chemical Composition of Natural Hydrolates and Their Antimicrobial Activity on Arcobacter-Like Cells in Comparison with Other Microorganisms. Molecules.

[4] Ramawat K.G., Mérillon J.M. (2013). Natural Products: Phytochemistry, Botany and Metabolism of Alkaloids, Phenolics and Terpenes.

[5] Diass K., Brahmi F., Mokhtari O., Abdellaoui S., Hammouti B. (2021). Biological and pharmaceutical properties of essential oils of *Rosmarinus officinalis* L. and *Lavandula officinalis* L. Mater. Today-Proc..

[6] Moisa C., Lupitu A., Pop G., Chambre D.R., Copolovici L., Cioca G., Bungau S., Copolovici D.M. (2019). Variation of the Chemical Composition of *Thymus Vulgaris* Essential Oils by Phenological Stages. Rev. Chim-Buchar..

[7] Chambre D.R., Moisa C., Lupitu A., Copolovici L., Pop G., Copolovici D.M. (2020). Chemical composition, antioxidant capacity, and thermal behavior of *Satureja hortensis* essential oil. Sci. Rep..

[8] Dutra T.V., Castro J.C., Menezes J.L., Ramos T.R., do Prado I.N., Machinski M., Mikcha J.M.G., Filho B.A.d.A. (2019). Bioactivity of oregano (*Origanum vulgare*) essential oil against *Alicyclobacillus* spp. Ind. Crops Prod..

[9] Ebadollahi A., Ziaee M., Palla F. (2020). Essential Oils Extracted from Different Species of the *Lamiaceae* Plant Family as Prospective Bioagents against Several Detrimental Pests. Molecules.

[10] Blažeković B., Yang W., Wang Y., Li C., Kindl M., Pepeljnjak S., Vladimir-Knežević S. (2018). Chemical composition, antimicrobial and antioxidant activities of essential oils of *Lavandula* × *intermedia* ‘*Budrovka*’ and *L. angustifolia* cultivated in Croatia. Ind. Crops Prod..

[11] De Oliveira A.A., França L.P., Ramos A.d.S., Ferreira J.L.P., Maria A.C.B., Oliveira K.M.T., Earle S.A., da Silva J.N., Branches A.D.S., Barros G.d.A. (2021). Larvicidal, adulticidal and repellent activities against Aedes aegypti L. of two commonly used spices, *Origanum vulgare* L. and *Thymus vulgaris* L. S. Afr. J. Bot..

[12] Kıvrak Ş. (2018). Essential oil composition and antioxidant activities of eight cultivars of Lavender and Lavandin from western Anatolia. Ind. Crops Prod..

[13] Gökdoğan O. (2016). Determination of input-output energy and economic analysis of lavender production in Turkey. Int. J. Agric. Biol..

[14] Rajeswara Rao B.R. (2013). Hydrosols and water-soluble essential oils: Their medicinal and biological properties. Recent Progress in Medicinal Plants.

[15] Ridder M. Essential Oils Market Worldwide—Statistics & Facts. https://www.statista.com/topics/5174/essential-oils/#dossierKeyfigures.

[16] Ridder M. Value of Lavender Oil Market Worldwide from 2014 to 2022. https://www.statista.com/statistics/973554/global-lavender-oil-market-value/.

[17] Patil S.M., Ramu R., Shirahatti P.S., Shivamallu C., Amachawadi R.G. (2021). A systematic review on ethnopharmacology, phytochemistry and pharmacological aspects of *Thymus vulgaris* Linn. Heliyon.

[18] Espíndola K.M.M., Ferreira R.G., Narvaez L.E.M., Silva Rosario A.C.R., da Silva A.H.M., Silva A.G.B., Vieira A.P.O., Monteiro M.C. (2019). Chemical and Pharmacological Aspects of Caffeic Acid and Its Activity in Hepatocarcinoma. Front. Oncol..

[19] Heidari Z., Salehzadeh A., Sadat Shandiz S.A., Tajdoost S. (2018). Anti-cancer and anti-oxidant properties of ethanolic leaf extract of *Thymus vulgaris* and its bio-functionalized silver nanoparticles. 3 Biotech..

[20] Salehi B., Mishra A.P., Shukla I., Sharifi-Rad M., Contreras M.d.M., Segura-Carretero A., Fathi H., Nasrabadi N.N., Kobarfard F., Sharifi-Rad J. (2018). Thymol, thyme, and other plant sources: Health and potential uses. Phytother. Res..

[21] Kiskó G., Roller S. (2005). Carvacrol and *p*-cymene inactivate *Escherichia coli* O157:H7 in apple juice. BMC Microbiol..

[22] Saibabu V., Fatima Z., Khan L.A., Hameed S. (2015). Therapeutic Potential of Dietary Phenolic Acids. Adv. Pharm. Sci..

[23] Arsenijević J., Drobac M., Šoštarić I., Ražić S., Milenković M., Couladis M., Maksimović Z. (2016). Bioactivity of herbal tea of Hungarian thyme based on the composition of volatiles and polyphenolics. Ind. Crops Prod..

[24] Arsenijević J., Drobac M., Šoštarić I., Jevđović R., Živković J., Ražić S., Moravčević Đ., Maksimović Z. (2019). Comparison of essential oils and hydromethanol extracts of cultivated and wild growing *Thymus pannonicus* All. Ind. Crops Prod..

[25] Lis-Balchin M.T., Peter K.V. (2012). 17—Lavender. Handbook of Herbs and Spices (Second Edition).

[26] Erland L.A.E., Mahmoud S.S., Preedy V.R. (2016). Chapter 57—Lavender (*Lavandula angustifolia*) oils. Essential Oils in Food Preservation, Flavor and Safety.

[27] Fascella G., D’Angiolillo F., Ruberto G., Napoli E. (2020). Agronomic performance, essential oils and hydrodistillation wastewaters of *Lavandula angustifolia* grown on biochar-based substrates. Ind. Crops Prod..

[28] Abou Baker D.H., Amarowicz R., Kandeil A., Ali M.A., Ibrahim E.A. (2021). Antiviral activity of *Lavandula angustifolia* L. and *Salvia officinalis* L. essential oils against avian influenza H5N1 virus. J. Sci. Food Agric..

[29] Détár E., Németh É.Z., Gosztola B., Demján I., Pluhár Z. (2020). Effects of variety and growth year on the essential oil properties of lavender (*Lavandula angustifolia* Mill.) and lavandin (*Lavandula* x *intermedia* Emeric ex Loisel.). Biochem. Syst. Ecol..

[30] Lafhal S., Bombarda I., Dupuy N., Jean M., Ruiz K., Vanloot P., Vanthuyne N. (2020). Chiroptical fingerprints to characterize lavender and lavandin essential oils. J. Chromatogr. A.

[31] Sałata A., Buczkowska H., Nurzyńska-Wierdak R. (2020). Yield, Essential Oil Content, and Quality Performance of *Lavandula angustifolia* Leaves, as Affected by Supplementary Irrigation and Drying Methods. Agriculture.

[32] Firoozeei T.S., Feizi A., Rezaeizadeh H., Zargaran A., Roohafza H.R., Karimi M. (2021). The antidepressant effects of lavender (Lavandula angustifolia Mill.): A systematic review and meta-analysis of randomized controlled clinical trials. Complement. Med..

[33] Ebrahimi H., Mardani A., Basirinezhad M.H., Hamidzadeh A., Eskandari F. (2021). The effects of Lavender and Chamomile essential oil inhalation aromatherapy on depression, anxiety and stress in older community-dwelling people: A randomized controlled trial. Explore.

[34] Şahin S., Tokgöz B., Demir G. (2021). Effect of Lavender Aromatherapy on Arteriovenous Fistula Puncture Pain and the Level of State and Trait Anxiety in Hemodialysis Patients: A Randomized Controlled Trial. Pain. Manag. Nurs..

[35] Abedian S., Abedi P., Jahanfar S., Iravani M., Zahedian M. (2020). The effect of Lavender on pain and healing of episiotomy: A systematic review. Complement. Med..

[36] Martínez-Gil A.M., Pardo-García A.I., Zalacain A., Alonso G.L., Salinas M.R. (2013). Lavandin hydrolat applications to Petit Verdot vineyards and their impact on their wine aroma compounds. Int. Food Res. J..

[37] Garzoli S., Turchetti G., Giacomello P., Tiezzi A., Laghezza Masci V., Ovidi E. (2019). Liquid and Vapour Phase of Lavandin (*Lavandula* × *intermedia*) Essential Oil: Chemical Composition and Antimicrobial Activity. Molecules.

[38] Cui H., Zhang C., Li C., Lin L. (2019). Antibacterial mechanism of oregano essential oil. Ind. Crops Prod..

[39] Martucci J.F., Gende L.B., Neira L.M., Ruseckaite R.A. (2015). Oregano and lavender essential oils as antioxidant and antimicrobial additives of biogenic gelatin films. Ind. Crops Prod..

[40] Radünz M., Mota Camargo T., Santos Hackbart H.C.d., Inchauspe Correa Alves P., Radünz A.L., Avila Gandra E., da Rosa Zavareze E. (2021). Chemical composition and in vitro antioxidant and antihyperglycemic activities of clove, thyme, oregano, and sweet orange essential oils. LWT.

[41] Ortega-Ramirez L.A., Rodriguez-Garcia I., Silva-Espinoza B.A., Ayala-Zavala J.F., Preedy V.R. (2016). Chapter 71—Oregano (*Origanum* spp.) oils. Essential Oils in Food Preservation, Flavor and Safety.

[42] Kintzios S.E., Peter K.V. (2004). Oregano. Handbook of Herbs and Spices.

[43] De Martino L., de Feo V., Formisano C., Mignola E., Senatore F. (2009). Chemical Composition and Antimicrobial Activity of the Essential Oils from Three Chemotypes of *Origanum vulgare* L. ssp. *hirtum* (Link) Ietswaart Growing Wild in Campania (Southern Italy). Molecules.

[44] Moisa C., Copolovici L., Pop G., Lupitu A., Ciutina V., Copolovici D. (2018). Essential Oil Composition, Total Phenolic Content, and Antioxidant Activity-Determined from Leaves, Flowers and Stems of *Origanum Vulgare* L. Var. Aureum. Sciendo.

[45] Jeannot V., Chahboun J., Russel D., Casabianca H. (2003). *Origanum compactum Bentham*: Composition of the hydrolat aromatic fraction, comparison with the essential oil and its interest in aromatherapy. Int. J. Aromather..

[46] Smail A., Badiaa L., Miguel M. (2011). Antioxidant activity of some Morrocan hydrosols. J. Med. Plant. Res..

[47] Paolini J., Leandri C., Desjobert J.-M., Barboni T., Costa J. (2008). Comparison of liquid-liquid extraction with headspace methods for the characterization of volatile fractions of commercial hydrolats from typically Mediterranean species. J. Chromatogr. A.

[48] Garneau L., Collin G., Gagnon H. (2014). Chemical composition and stability of the hydrosols obtained during essential oil production. I. The case of *Melissa officinalis* L. and *Asarum canadense* L. Am. J. Essent. Oil..

[49] Labadie C., Ginies C., Guinebretiere M.-H., Renard C.M.G.C., Cerutti C., Carlin F. (2015). Hydrosols of orange blossom (*Citrus aurantium*), and rose flower (*Rosa damascena* and *Rosa centifolia*) support the growth of a heterogeneous spoilage microbiota. Int. Food Res. J..

[50] Clarke S., Clarke S. (2008). Chapter 3—Families of compounds that occur in essential oils. Essential Chemistry for Aromatherapy.

[51] Turek C., Stintzing F.C. (2013). Stability of Essential Oils: A Review. Compr. Rev. Food Sci. Food Saf..

[52] Nikolić M., Glamočlija J., Ferreira I.C., Calhelha R.C., Fernandes Â., Marković T., Marković D., Giweli A., Soković M. (2014). Chemical composition, antimicrobial, antioxidant and antitumor activity of *Thymus serpyllum* L., *Thymus algeriensis Boiss.* and *Reut* and *Thymus vulgaris* L. essential oils. Ind. Crops Prod..

[53] Moukhles A., Mansour A., Ellaghdach A., Abrini J. (2018). Chemical composition and in vitro antibacterial activity of the pure essential oils and essential oils extracted from their corresponding hydrolats from different wild varieties of Moroccan thyme. J. Mater. Env. Sci..

[54] Hay Y.-O., Sierra M., Sequeda-Castañeda L., Bonnafous C., Delgado Raynaud C. (2018). Evaluation of combinations of essential oils and essential oils with hydrosols on antimicrobial and antioxidant activities. J. Pharm. Pharm..

[55] Arsenijević J., Marković J., Šoštarić I., Ražić S. (2013). A chemometrics as a powerful tool in the elucidation of the role of metals in the biosynthesis of volatile organic compounds in Hungarian thyme samples. Plant. Physiol. Biochem..

[56] Garzoli S., Laghezza Masci V., Franceschi S., Tiezzi A., Giacomello P., Ovidi E. (2021). Headspace/GC-MS Analysis and Investigation of Antibacterial, Antioxidant and Cytotoxic Activity of Essential Oils and Hydrolates from *Rosmarinus officinalis* L. and *Lavandula angustifolia* Miller. Foods.

[57] Węglarz Z., Kosakowska O., Przybył J.L., Pióro-Jabrucka E., Bączek K. (2020). The Quality of Greek Oregano (*O. vulgare* L. subsp. *hirtum* (Link) Ietswaart) and Common Oregano (*O. vulgare* L. subsp. *vulgare*) Cultivated in the Temperate Climate of Central Europe.. Foods.

[58] Gharib F.A.E.-L., Silva J.T.d. (2012). Composition, Total Phenolic Content and Antioxidant Activity of the Essential Oil of Four *Lamiaceae* Herbs. Med. Aromat. Plant. Sci. Biotechnol..

[59] Floegel A., Kim D.-O., Chung S.-J., Koo S.I., Chun O.K. (2011). Comparison of ABTS/DPPH assays to measure antioxidant capacity in popular antioxidant-rich US foods. J. Food Compos. Anal..

[60] Dawidowicz A.L., Olszowy M. (2013). The importance of solvent type in estimating antioxidant properties of phenolic compounds by ABTS assay. Eur. Food Res. Technol..

[61] Csakvari A.C., Moisa C., Radu D.G., Olariu L.M., Lupitu A.I., Panda A.O., Pop G., Chambre D., Socoliuc V., Copolovici L. (2021). Green Synthesis, Characterization, and Antibacterial Properties of Silver Nanoparticles Obtained by Using Diverse Varieties of *Cannabis sativa* Leaf Extracts. Molecules.

[62] De Souza W.F.C., de Lucerna F.A., de Castro R.J.S., de Oliviera C.P., Quirino M.R., Martins L.P. (2021). Exploiting the chemical composition of essential oils from *Psidium cattleianum* and *Psidium guajava* and its antimicrobial and antioxidant properties. J. Food Sci..

[63] Zhu J.-J., Yang J.-J., Wu G.-J., Jiang J.-G. (2020). Comparative antioxidant, anticancer and antimicrobial activities of essential oils from *Semen Platycladi* by different extraction methods. Ind. Crops Prod..

[64] Adams R.P. (2017). Identification of Essential Oil Components by Gas Chromatography/Mass Spectrometry. Allured Pub. Corp..

[65] Moisă C., Copolovici L., Bungau S., Pop G., Imbrea I., Lupitu A.-I., Nemeth S., Copolovici D. (2018). Wastes resulting from aromatic plants distillation—Bio-sources of antioxidants and phenolic compounds with biological active principles. Farmacia.

[66] Bogdan M.A., Bungau S., Tit D.M., Zaha D.C., Nechifor A.C., Behl T., Chambre D., Lupitu A.I., Copolovici L., Copolovici D.M. (2021). Chemical Profile, Antioxidant Capacity, and Antimicrobial Activity of Essential Oils Extracted from Three Different Varieties (Moldoveanca 4, Vis Magic 10, and Alba 7) of *Lavandula angustifolia*. Molecules.

